# Lateral approach for total knee arthroplasty in patients with valgus deformity: A step-by-step surgical technique

**DOI:** 10.1051/sicotj/2025047

**Published:** 2025-09-01

**Authors:** Bin Yang, Christos Koutserimpas, Bin Sun, Cécile Batailler, Elvire Servien, Sébastien Lustig

**Affiliations:** 1 Orthopaedics Surgery and Sports Medicine Department, FIFA Medical Center of Excellence, Croix-Rousse Hospital, Hospices Civils de Lyon, Lyon North University Hospital 103 Grande Rue de la Croix-Rousse 69004 Lyon France; 2 Joint Surgery Group, Department of Orthopaedics, Peking University International Hospital 102206 Beijing China; 3 School of Rehabilitation Health Sciences, University of Patras, Campus of University of Patras, Rio 26504 Patras Greece; 4 Univ Lyon, Claude Bernard Lyon 1 University, IFSTTAR, LBMC UMR_T9406 25 Avenue François Mitterand 69622 Lyon France; 5 LIBM-EA 7424, Interuniversity Laboratory of Biology of Mobility, Claude Bernard Lyon 1 University 43 Bd du 11 Novembre 1918 69100 Villeurbanne Lyon France

**Keywords:** Total knee replacement, Knee anatomy, Knee reconstruction, Arthroplasty, Surgical approach

## Abstract

*Background*: The lateral approach in total knee arthroplasty (TKA) is indicated primarily for patients with valgus knee deformity, as it allows direct access to the lateral anatomy and systematic correction of associated pathologies. *Surgical Technique*: This technique involves strategic lateral soft tissue releases, which improve exposure to the posterolateral corner, enhance tibial rotation, and support patellar alignment without compromising medial vascularity or requiring a tibial tubercle osteotomy for joint exposure. Critical steps in the lateral TKA approach include maintaining a capsular-synovial overlap and preserving the Hoffa fat pad for optimal joint closure, releasing the lateral soft-tissue structures, and using a contralateral tibial cutting guide for enhanced access and protection of the patellar tendon. *Discussion*: These techniques collectively allow for a balanced, stable joint with effective alignment and soft tissue management. Outcomes of the lateral approach in valgus TKA are comparable to those of the medial approach, with similar functional outcomes, range of motion, and surgical time. Some studies even report superior patellar tracking and function scores with the lateral approach. Complication rates are low, though attention is required to avoid peroneal nerve injury in severe deformities. Future research involving large, randomized controlled trials is recommended to substantiate these favorable outcomes and guide long-term treatment strategies for valgus TKA.

## Introduction

Traditionally, the medial approaches are used for total knee arthroplasty (TKA) [1, 2]. In valgus TKA, the medial approach offers limited access to the pathologic anatomy and presents technical challenges, including restricted posterolateral exposure, increased tibial external rotation, compromised lateral vascularity, and risk of excessive lateral release [3, 4] (Table 1). In contrast, the lateral approach allows direct access to valgus pathology, enabling efficient lateral release through exposure, improved posterolateral access via tibial internal rotation, and sequential gap balancing [1]. It also preserves medial vascularity, ensures adequate soft tissue management, optimizes patellar tracking, and supports rehabilitation by maintaining medial quadriceps integrity [2].

**Table 1:** Comparison of commonly used surgical approaches for total knee arthroplasty (TKA), highlighting their respective advantages and disadvantages.

Approach for TKA	Advantages	Disadvantages
Anterior Midline (Medial Parapatellar)	Most commonly used, familiar to most surgeons Excellent exposure Versatile	Potential disruption of the extensor mechanism May affect patellar tracking
Midvastus	Preserves the quadriceps tendon Potential for quicker early recovery Improved patellar tracking	Technically more demanding Limited exposure in obese or muscular patients
Subvastus	True quadriceps-sparing approach Faster rehabilitation and reduced postoperative pain	Limited exposure, especially in stiff or valgus knees Not suitable for all patients (severe valgus cases)
Lateral	Direct access to lateral pathology in valgus knees Facilitates sequential lateral release Preserves medial soft tissues	Limited access to posteromedial structures Learning curve Contraindicated with previous scars or in varus knees

This surgical technique article outlines a step-by-step, structured approach to TKA using the lateral approach in patients with valgus deformity. Key technical steps, indications, and tips for optimal exposure and closure are outlined to assist in managing these cases effectively.

## Surgical technique

Table 2 and Video 1 provide a step-by-step guide for the lateral approach in TKA.

**Table 2:** Step-by-step technique of the lateral approach for total knee arthroplasty in valgus knees.

Step-by-step surgical technique	
Patient setting and positioning	1. Patient in supine position.
	2. With or without a tourniquet.
	3. Position the knee in approximately 90 degrees of flexion.
Incision and exposure	4. Lateral parapatellar approach.
	5. Two-layer capsulotomy.
	6. Leave a 1 cm overlap between the two layers of joint capsule for final capsular closure.
Hoffa pad management	7. Dissect the Hoffa fat pad from the underside of the patellar tendon, releasing its medial attachment while preserving its attachment to the lateral retinaculum and maintaining blood supply. Retention of the Hoffa fat pad may facilitate closure of the lower retinacular incision, particularly after substantial correction of a valgus deformity.
Joint access	8. Revert the patella with the knee in extension, then flex the knee to 90 degrees.
	9. Adequate exposure can be achieved without the need for tibial tubercle osteotomy.
Bone cuts and preparation	10. Measure the gap between the distal femoral cutting guide and the lateral femoral condyle (caused by dysplasia or wear) to match later during gap balancing with the posterior lateral femoral condyle.
	11. After completing the distal femoral cut, place the femoral guide aligned with the Whiteside line. Use an osteotome to match the previously measured gap between the distal femoral and posterior lateral femoral condyles.
	12. To perform the tibial cut, utilize a contralateral cutting guide to protect the patellar tendon and facilitate access across the tibia.
	13. Perform the cuts using an intramedullary guide for the femur and intra- or extramedullary guides for the tibia.
	14. Shift the tibial cutting guide proximally by 2 mm to account for the medial plateau’s lower reference point compared to the lateral plateau.
	15. Maximize knee flexion, anteriorly sublux the tibia, and externally rotate it to expose the posteromedial tibial plateau.
	16. Thoroughly remove osteophytes, including those on the posteromedial side, and excise the menisci.
Implantation of the prostheses	17. Ensure clear visualization of the posteromedial tibial plateau to avoid excessive lateral rotation of the tibial component.
	18. Pay special attention to the mediolateral positioning of the femoral component, as the lateral approach may increase the risk of misalignment for surgeons accustomed to a medial approach.
	19. Perform patellar resurfacing as appropriate based on patient characteristics and surgeon preference.
	20. Confirm gap balancing, overall alignment, and perform appropriate soft tissue releases.
	21. Position the final implants.
Closure	22. Close the joint with the knee flexed at 90 degrees. Close the angular position first, then proceed proximally. Use the overlapped two layers of capsule proximal and any remaining fat pad distally to reinforce joint closure, creating a “tight seal” for the knee joint. Finally, excise the excess fat pad.

### Positioning

The patient is placed in the supine position with a lateral support at the thigh and a foot bolster to stabilize the limb. The surgical leg is prepared and draped in a sterile manner, with care taken to allow for a full range of motion during the procedure. Standard prophylactic antibiotics are administered before incision.

### Skin incision and initial exposure

**Figure 1:**
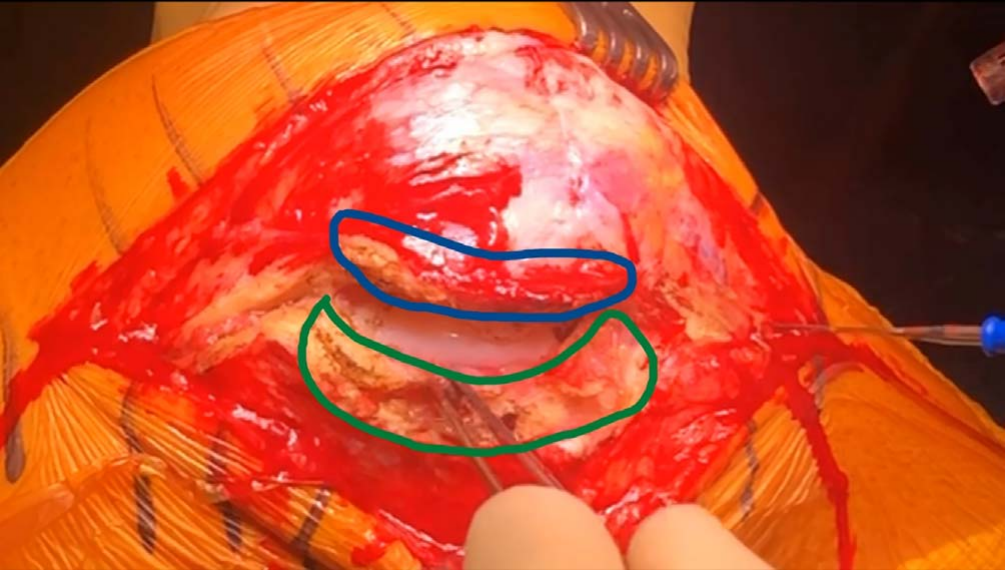
Exposure with the lateral approach. A lateral parapatellar approach with a two-layer capsulotomy, leaving a 1 cm overlap, is used.

A lateral parapatellar incision is performed, beginning approximately 2–3 cm proximal to the patella and extending distally toward the tibial tubercle. A two-layer capsulotomy is executed: the initial superficial cut is made on the side away from the patella, followed by dissection inward, and finalized with a deep incision adjacent to the patella. This sequence preserves capsular integrity and facilitates secure closure. A 1 cm overlap of the lateral joint capsule is intentionally left to enable anatomic reapproximation during closure (Figure 1).

### Soft tissue handling and Hoffa fat pad management

**Figure 2:**
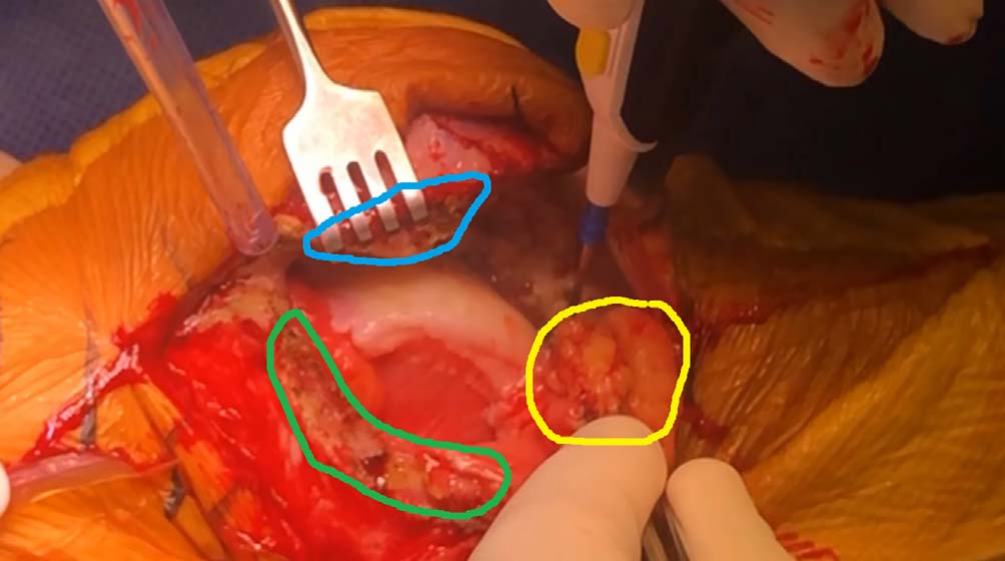
The lateral parapatellar approach involves a two-layer capsulotomy, leaving a 1 cm tissue overlap on both sides of the incision (green and blue areas) for secure closure, while preserving as much fat pad as possible behind the patellar tendon (yellow area) to facilitate capsular closure.

Preservation of the Hoffa fat pad is prioritized to assist in closure of the distal portion of the incision, especially after significant valgus correction. The fat pad is carefully dissected from the undersurface of the patellar tendon, minimizing unnecessary excision to maintain structural support and facilitate joint sealing (Figure 2).

### Patellar management and tibial access

**Figure 3:**
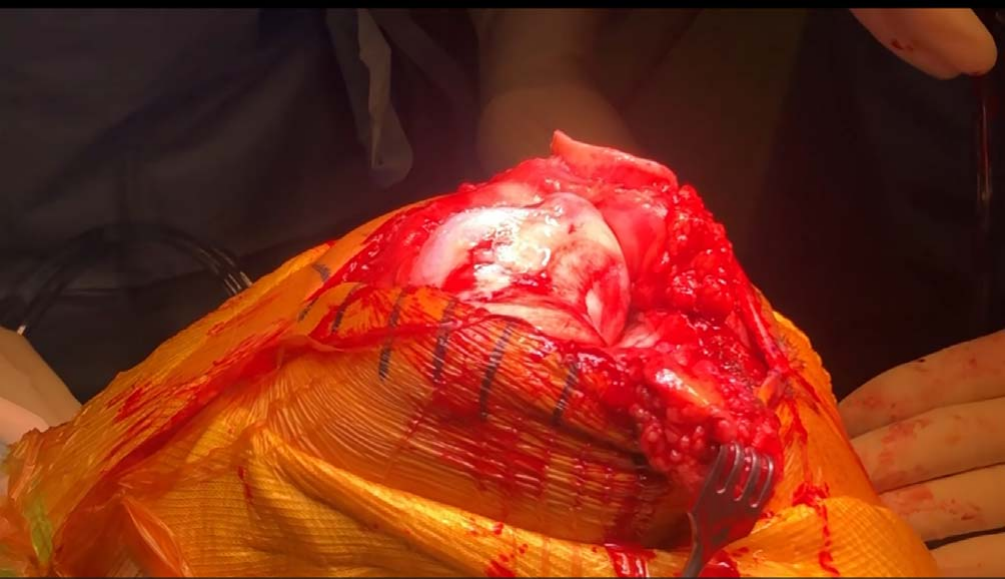
Exposure of the knee joint after reverting the patella with the knee in extension, then flexing the knee to 90 degrees.

The patella is everted with the knee in extension, followed by flexion to 90 degrees, to facilitate deep exposure (Figure 3). This maneuver allows direct visualization and access to the lateral femoral condyle and posterolateral tibia.

### Bone preparation

**Figure 4:**
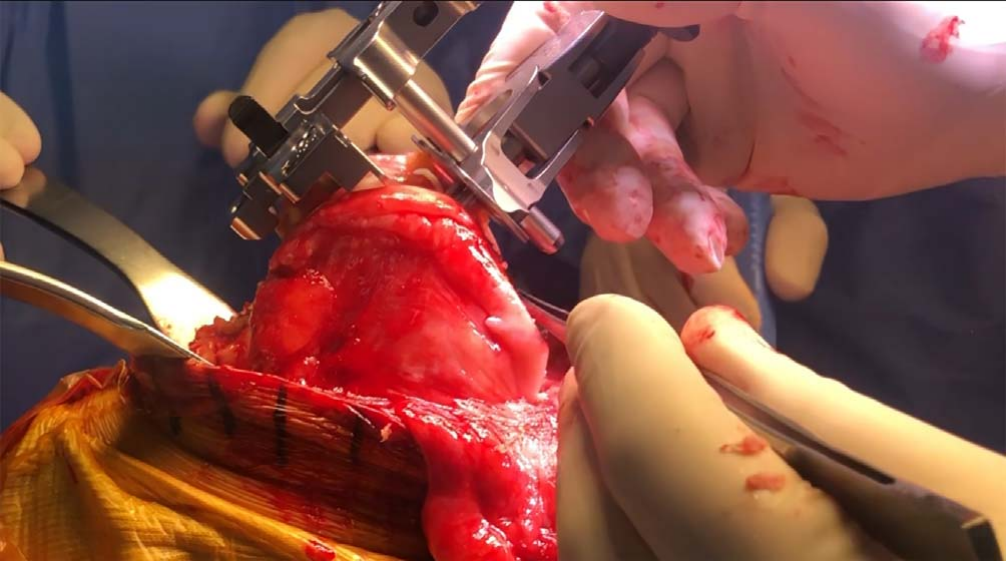
Key steps during the main procedure: Measurement of the gap between the distal femoral cutting guide and the lateral femoral condyle.

**Figure 5:**
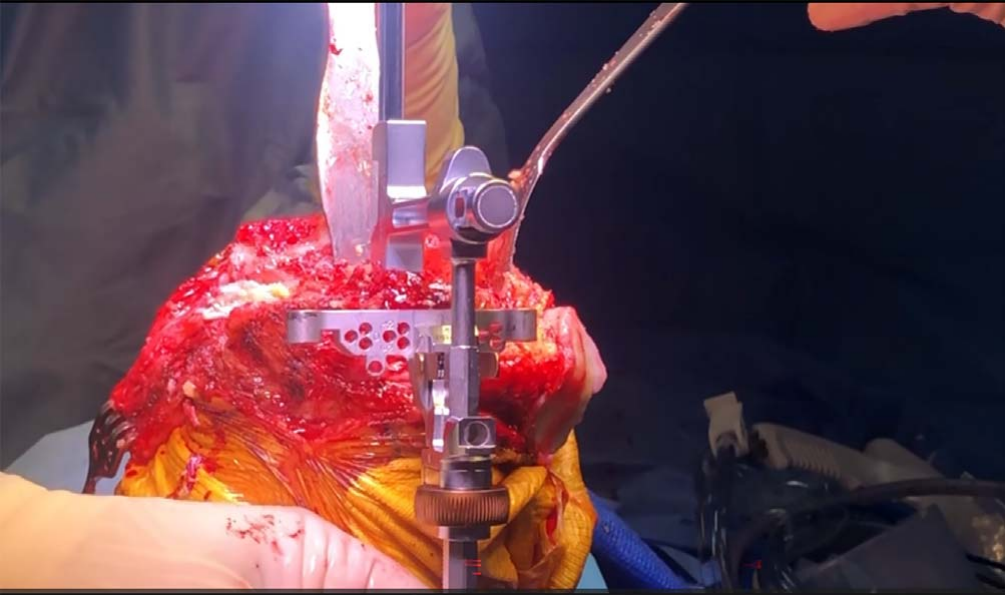
A contralateral cutting guide to perform the tibial cut is utilized for better access of the cutting saw without the risk of injuring the patellar tendon.

During the femoral preparation, the gap between the distal femoral cutting guide and the lateral femoral condyle, often altered due to dysplasia or wear, is measured to guide subsequent posterior condylar balancing (Figure 4). After completing the distal femoral cut, the femoral guide is aligned with the Whiteside line, and an osteotome is used to recreate the previously measured gap posteriorly. A contralateral tibial cutting guide is employed to improve access and protect the patellar tendon during tibial preparation (Figure 5). Bone cuts are performed using an intramedullary guide for the femur and intra- or extramedullary alignment for the tibia. The tibial guide is shifted proximally by 2 mm to compensate for the lower medial plateau reference. Flexion of the knee is maximized, and the tibia is subluxed anteriorly and externally rotated to expose the posteromedial plateau. Osteophytes are thoroughly removed, particularly posteromedially, and both menisci are excised.

### Sequential lateral releases

The tight lateral structures, including the lateral capsule, iliotibial band, and vastus lateralis tendon, are sequentially released based on intraoperative assessment of gap balancing. In rare cases with fixed deformity, additional release of the lateral collateral ligament, popliteus tendon, or lateral gastrocnemius may be required. These structures are accessed efficiently through the lateral approach, and releases are titrated to restore coronal alignment and ensure stability.

### Medial exposure considerations

**Figure 6:**
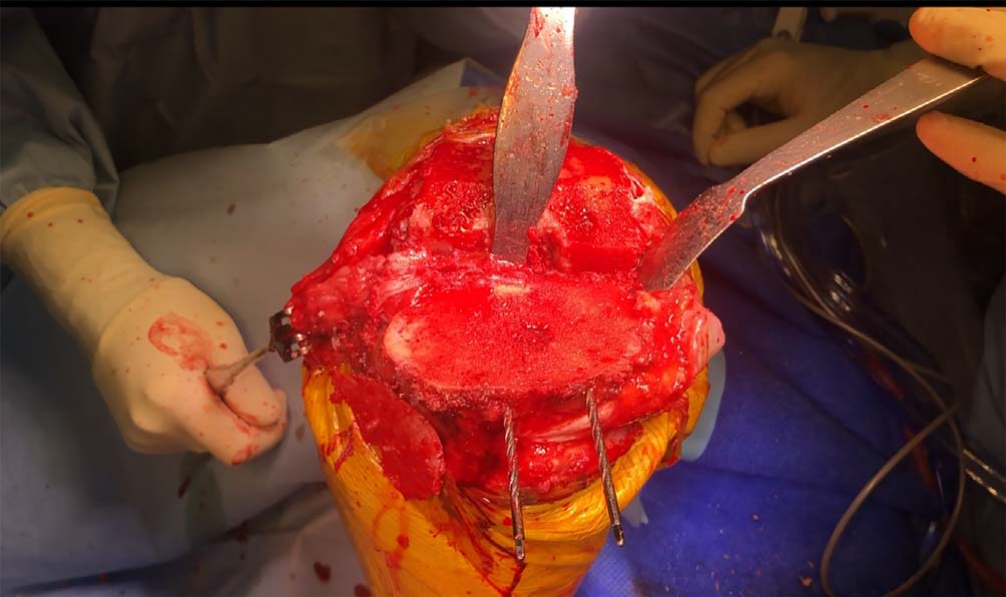
Exposure of the posteromedial tibial plateau.

Despite limited access to the posteromedial side, this approach provides sufficient visualization for bone preparation without requiring tibial tubercle osteotomy. In most cases, adequate medial exposure is achieved without additional extensile maneuvers (Figure 6).

### Implantation and closure

**Figure 7:**
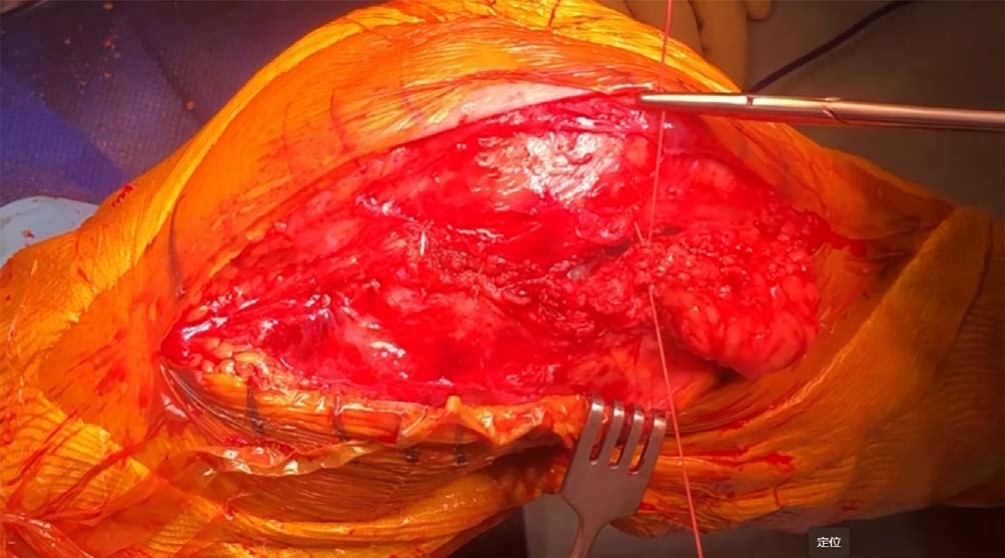
Closure of the lateral approach: Any remaining fat pad distally is used to reinforce joint closure.

Once the bone cuts and trial confirm satisfactory balance and alignment, the definitive prosthetic components are implanted. Closure begins distally in the angular position and proceeds proximally. The overlapping layers of the capsule are sutured meticulously, reinforced by the retained distal fat pad, which is folded into the closure for additional support. Any redundant portions of the fat pad are trimmed to avoid bulk beneath the skin (Figure 7).

## Discussion

This surgical technique aims to provide a reproducible and effective method for performing TKA in patients with valgus deformity using the lateral approach. The lateral approach offers several advantages in managing valgus pathology, including direct access to contracted lateral structures, preservation of medial soft tissues, and improved control of patellar tracking. To optimize the outcomes of this technique, several critical steps have been emphasized. First, a 1 cm overlap of the lateral joint capsule is deliberately maintained to facilitate reliable capsular closure following deformity correction. This is achieved by incising the capsule in a stepwise manner, starting superficially on the side opposite the patella, progressing through careful dissection, and finishing with a deep incision adjacent to the patella, to avoid misalignment during closure. Second, preservation of the Hoffa fat pad is recommended, particularly distally, as it can be instrumental in achieving a tension-free closure after valgus correction. Third, the use of a contralateral tibial cutting guide improves access to the tibial plateau and protects the patellar tendon during bone preparation.

The primary indication for the lateral approach in TKA is the presence of a valgus deformity. Fixed valgus deformity often presents with unique anatomical challenges, such as lateral femoral condyle hypoplasia, femorotibial malrotation, resorption of the lateral femoral condyle and tibial plateau, and an enlarged medial condyle [5–7]. Tight lateral structures and a frequently deformed, subluxed patella over the lateral condyle are common features [8]. Soft tissue deficiencies in this region can pose challenges for achieving adequate prosthetic coverage and joint seal. Correcting fixed contractures in valgus TKA typically involves a sequence of potential releases, including the lateral capsule, iliotibial band (ITB), vastus lateralis tendon, and, in rare cases, the lateral collateral ligament (LCL), popliteus, lateral gastrocnemius, and fibular head (where the LCL is preserved and lengthened) [8, 9]. These releases are most effectively achieved through direct lateral access.

This approach has few contraindications. It may be unsuitable if a previous scar is located within 6–8 cm of the incision site, as the skin bridge between them could be at risk of compromised viability [2, 5]. Additionally, due to the limited access to the posteromedial side of the knee in comparison to medial approaches, this technique is generally not recommended for cases with varus deformity, as releasing medial contractures can be challenging from the lateral side [2, 10]. Furthermore, in valgus cases where the axial deformity is greater than 20°, and the medial stabilizers are not functional, if a medial capsular plication is planned, then the lateral approach should not be used.

The choice of surgical approach for TKA in valgus knee remains a topic of debate. Key technical elements, including surgical exposure, bone cuts, and ligament balancing, are essential to achieving proper alignment and stability. Selecting the appropriate surgical approach is critical and forms the foundation of effective preoperative planning. Recent meta-analyses have shown that patients undergoing TKA with a medial approach demonstrated, on average, comparable postoperative Knee Society Scores (KSS) and flexion range of motion (ROM) to those treated with a lateral approach [1, 7]. Both approaches showed similar surgical times, postoperative hip-knee-ankle (HKA) angles, KSS function scores, and complication rates [1, 7]. Furthermore, another meta-analysis by Xu et al. revealed higher KSS and KSS function scores in patients treated with the lateral approach, while both the lateral and medial approaches showed comparable results in surgical time, range of motion (ROM), correction of valgus knee deformity, and overall complication rates [6]. The lateral approach in valgus knees can also be utilized in image-based robotic TKA, without problems during pins’ insertion and the workflow of the surgery [11–13].

With adequate experience, the risk of complications with this approach is minimal. Component alignment has been consistently accurate in various studies, and patellar tracking is optimized, reducing the likelihood of patellar instability. However, peroneal palsy remains a risk, especially in severe fixed valgus deformities. Excessive retraction or unintentional dissection may heighten the risk to the peroneal nerve. Hematoma, a potential source of compressive neuropathy, can be minimized with proper hemostasis [14]. Postoperative complications may also include difficulties in joint capsule closure, postoperative effusion, and poor incision healing [7, 14].

## Conclusion

The lateral approach for TKA in valgus deformity offers safe and reproducible access to the pathologic anatomy, facilitating effective alignment and patellar tracking without the need for tibial tubercle osteotomy. Further studies are warranted to confirm its long-term outcomes across broader patient populations.

## Data Availability

Data is available upon reasonable request to the corresponding author.

## Supplementary material

*Video 1*: The step-by-step technique for the lateral approach for total knee arthroplasty is presented. The supplementary material of this article is available at https://www.sicot-j.org/10.1051/sicotj/2025047/olm.
